# Progress in loop-mediated isothermal amplification assay for detection of *Schistosoma mansoni* DNA: towards a ready-to-use test

**DOI:** 10.1038/s41598-019-51342-2

**Published:** 2019-10-14

**Authors:** J. García-Bernalt Diego, P. Fernández-Soto, B. Crego-Vicente, S. Alonso-Castrillejo, B. Febrer-Sendra, A. Gómez-Sánchez, B. Vicente, J. López-Abán, A. Muro

**Affiliations:** Infectious and Tropical Diseases Research Group (e-INTRO), Biomedical Research Institute of Salamanca-Research Centre for Tropical Diseases at the University of Salamanca (IBSAL-CIETUS), Faculty of Pharmacy, University of Salamanca, Salamanca, Spain

**Keywords:** Parasitic infection, PCR-based techniques

## Abstract

Schistosomiasis is one of the most prevalent Neglected Tropical Disease, affecting approximately 250 million people worldwide. *Schistosoma mansoni* is the most important species causing human intestinal schistosomiasis. Despite significant efforts in recent decades, the global disease burden of schistosomiasis remains extremely high. This could partly be attributed to the absence of accurate diagnostic tools, primarily in endemic areas. Loop-mediated isothermal amplification (LAMP) is increasingly used in molecular diagnostics as a field-friendly alternative to many other complex molecular methods and it has been proposed as an ideal candidate for revolutionizing point-of-care molecular diagnostics. In a previous work, a LAMP-based method to detect *S*. *mansoni* DNA (SmMIT-LAMP) was developed by our research group for early diagnosis of active schistosomiasis in an experimental infection murine model. The SmMIT-LAMP has been further successfully evaluated in both human stool and snail samples and, recently, in human urine samples. In this study, we developed an important improvement for SmMIT-LAMP molecular assay, transforming it into a cold maintenance dry format suitable for potentially manufacturing as kit for ready-to-use for schistosomiasis diagnosis. This procedure could be applied to create dry LAMP kits for a laboratory setting and for diagnostic applications for other neglected tropical diseases.

## Introduction

The World Health Organization (WHO) identifies Neglected Tropical Diseases (NTD) as a diverse group of communicable chronic, debilitating and often stigmatizing infectious diseases affecting more than one thousand million people in tropical and subtropical regions along 149 countries, especially in populations living in extreme poverty and inadequate sanitation^1^. One of these NTD is schistosomiasis, a parasitic disease caused by trematode worms (blood flukes) of the genus *Schistosoma*. There are two major forms of human schistosomiasis: urogenital schistosomiasis, caused by *Schistosoma haematobium*, and intestinal schistosomiasis, caused by any of the organisms *S*. *mansoni*, *S*. *intercalatum*, *S*. *guineensis*, *S*. *japonicum*, and *S*. *mekongi*. It is estimated that 779 million people live within high-risk-of-infection areas and 250 million are infected, more than 80% of them living in sub-Saharan Africa^2^. The disease accounts for an estimated 1.9 million disability-adjusted life years (DALYs) annually^3^. In recent years, autochthonous outbreaks of urogenital schistosomiasis in the south of Europe have been reported^4^. All these figures emphasize the importance and the need for control and elimination of schistosomiasis. Despite significant efforts in recent decades, the global disease burden of schistosomiasis remains extremely high since a regular treatment with praziquantel, provision of water, sanitation and hygiene, new complimentary drugs, local recommendations for snail control, surveillance and management of hotspots, and long-term, well-structured control programmes are still needed in endemic areas. Besides, more accurate diagnostic tools for detecting schistosome infections are also required to help in the overall control of schistomiasis.

The traditional Kato-Katz fecal microscopic examination for counting schistosome eggs and immunology-based analyses detecting schistosome-derived circulating anodic (CAA) and cathodic (CCA) antigens mainly lack sensitivity in low-intensity infections and posttreatment conditions^5,6^. Antibody detection also lacks specificity, causing a high level of cross-reactivity^6^. Several PCR-based molecular approaches –conventional PCR (cPCR), real-time quantitative PCR (qPCR), droplet digital PCR (ddPCR)- have been proven effective in detection of schistosome-derived DNA with more sensitivity than parasitological and serological methods, especially in chronic infections^7^ and in low transmission areas^8^. However, diagnostic assays based on PCR are not widely used in resource-limited settings because they are time-consuming, complex, and require skilled personnel and expensive equipment^9^. Hence, the development of novel diagnostics methods for NTD complying with features, such us low cost, rapidity, usable with different kinds of samples, simple operation and interpretation, and detection capability with high sensitivity and specificity are vital to address the present limitations in use of PCR-based tests in low-income countries.

In recent years, a number of nucleic acid isothermal amplification techniques trying to fulfill these requirements have been developed^10^. Among the most promising is the loop-mediated isothermal amplification (LAMP) assay^11^. LAMP was initially reported in 2000 as a single tube method for rapid nucleic acids amplification under isothermal conditions with high specificity and efficiency^12^, allowing the direct visual discrimination of positive results^13^. Since then, LAMP technology has been successfully used for detection of many infectious agents in both animals and plants^14^. Nowadays, of the 20 NTD recognized by WHO (http://www.who.int/neglected_diseases/diseases/en/), LAMP reactions for 16 of them have been reported, including schistosomiasis^11^. The cheapness and user-friendliness of LAMP method, together with the increasing development of miniaturized instrument-free LAMP systems as lab-on-chip, makes it a cost-effective and simple tool that provides an alternative to PCR assays for low-cost chip-based point of care (POC) diagnostic applications in low-resource settings^15–18^. Even so, currently micro-LAMP systems for POC diagnostics still have limitations and do not completely satisfy WHO criteria of equipment-free/electricity-free operation^17^. One of the major obstacles for the application of LAMP in NTD-endemic countries has been the difficulty in maintaining the cold-chain to preserve reagents. Thus, the challenge is to develop a LAMP kit in a ready-to-use format with dried reagents useful for quick and simple application in field conditions. LAMP kits for Tuberculosis and Malaria are quite developed and already commercialized (available in Human Diagnostic Worldwide; https://www.human.de/products/molecular-dx/). Regarding NTD, the only LAMP prototype kits in dried form developed are for Chagas disease, Human African Trypanosomiasis^19^, and for multiplexing Dengue and Chikungunya viral infections^20^.

The two most common procedures used to stabilize pre-mixed LAMP reagents in a dried kit format are drying^21^ and lyophilization^22,23^. Lyophilization protocols allow all LAMP reagents to be initially included^20,22,24,25^, while in drying protocols at least a two-step dry-up is required, keeping the remaining components on ice for final addition to the mix at the time to run LAMP reaction^21,26^. In both cases, experimentally processes are laborious and require sophisticated equipment.

In general, stabilization protocols of reagents by drying are based on the use of molecules like trehalose, due to its ability to resist extreme dehydration and temperature conditions^27^. Besides, since it is produced naturally by a number of microorganisms, it is easy and cheap to obtain^28^. The stabilizing capacity of trehalose is a result of its physical properties*:* (*i*) interacts directly with the compound and protect it during the drying process and, (*ii*) limits the mobility of the compound increasing their state of hydration^29^. It has been proven that trehalose is useful for the preservation of reagents (including primers, dNTPs and enzymes) for PCR^30–32^ and LAMP^21^. Besides this application, it has been demonstrated to improve the analytical performance of other isothermal nucleic acid amplification methods, such as the exponential amplification reaction (EXPAR)^33^.

A LAMP method for detection of *S*. *mansoni* DNA (so called, SmMIT-LAMP) was previously established by our group for early diagnosis of active schistosomiasis in experimental infection in a murine model^34^. Recently, the SmMIT-LAMP was successfully evaluated in both human stool and snail samples from a low-transmission schistosomiasis-endemic area in Brazil^35^ and, even more recently, also in human urine samples^36^.

Here, in order to improve the established LAMP technique for *S*. *mansoni* DNA detection, we report the development of a desiccation procedure to stabilize the SmMIT-LAMP reagents in a single tube for conventional or real-time potential ready-to-use application in diagnosis of schistosomiasis *mansoni*.

## Material and Methods

### DNA extraction for molecular analysis

#### Schistosoma mansoni DNA

Genomic *S*. *mansoni* DNA was extracted from frozen adult worms available in our laboratory using NucleoSpin Tissue kit (MACHEREY-NAGEL, Germany) following manufacturers’ instructions. The concentration was measured using a NanoDrop (ND-1000; THERMO FISHER SCIENTIFIC, USA) and diluted to a final concentration of 0.5 ng/μL to be used as positive control (2 μL; 1 ng) in all LAMP trials.

#### DNA from patients’ tissue samples

DNA from 3 patients’ tissue samples with microscopy-confirmed infection with *S*. *mansoni* was tested by LAMP: cutaneous and hepatic biopsies (kindly provided by the University Hospital Vall d’Hebron, Barcelona, Spain) and an appendix biopsy (kindly provided by the Department of Parasitology, National Centre for Microbiology, Institute of Health Carlos III, Majadahonda, Madrid, Spain). DNA was isolated from tissue samples using the QIAamp DNA Mini Kit (QIAGEN, Hilden, Germany) following manufacturers’ instructions and then stored at −20 °C until use in LAMP reactions. The tissue samples were collected from adult patients attending at Hospitals as part of public health diagnostic activities. They signed an informed consent form.

### Conventional SmMIT-LAMP

Fresh conventional LAMP reactions to amplify *S*. *mansoni* DNA were performed using the set of four primers and conditions previously described by Fernández -Soto *et al*.^34^. In brief, in a volume of 25 μL were mixed: betaine (1 M) (SIGMA, USA); Isothermal Amplification Buffer 1X, supplementary MgSO_4_ (6 mM) and 8U of *Bst* polymerase 2.0 WarmStart (NEW ENGLAND BIOLABS, UK); dNTPs (1,4 mM each); FIP/BIP (40 pmol/μL each) and F3/B3 (5 pmol/μL each) (BIORON). The cLAMP reactions were carried out in a Dry-Bath (No. DB-006) at 65 °C for 1 h followed up by 10 min at 80 °C to stop amplification. Amplification products were visualized with naked eye by adding 2 μL of SYBR Green I 1000× (INVITROGEN) in each post-amplified tube. LAMP products were also monitored on 1.5% agarose gels when required.

### Real-time SmMIT-LAMP

Each 25 μL liquid freshly prepared real-time LAMP reaction to amplify *S*. *mansoni* DNA contained the same reagents as those for conventional SmMIT-LAMP together with 0.40 μL of EvaGreen 20X in water (BIOTIUM) to monitor the fluorescence over time. In some trials, betaine was removed from reaction mixes. The real-time SmMIT-LAMP reactions were performed in 8-tube Genie Strips on a portable Genie III device (OPTIGENE Ltd., Horsham, UK) at 65 °C for 60 min followed up by 10 min at 80 °C. Amplicons were confirmed on 1.5% agarose gels when required.

### Stabilization procedures of LAMP reagents for conventional and real-time tests

To carry out the stabilization of the conventional SmMIT-LAMP mixtures we based our trials on a previous protocol described by Hayasida *et al*.^21^ that uses a drying procedure with trehalose. Briefly, this two-step protocol consisted in: first, primers and dye (“colori-fluorometric indicator”, CFI: a mix of hydroxyl-naphtol blue and GelGreen dissolved in distilled water) were air dried in the presence of 0,56 μL of trehalose (2 M) and 0,14 μL of 50% glycerol for 30 min under a flow of clean air in the top of a 0.2 mL microtube. Secondly, *Bst* polymerase and dNTPs were dried in the presence of additional 1.5 μL of trehalose for 15 min. Finally, reaction mixes were further dried over 24 h under a vacuum in a container with P_2_O_5_ and silica gel, and then stored in an aluminium bag with zeolite molecular sieves.

Based on the above, we tried to simplify this drying protocol by subjecting the SmMIT-LAMP reagents to vacuum process using: (1) centrifugation (so called, concentration) or (2) without centrifugation (so called, desiccation) following two or single dry-up steps as shown in Supplementary Fig. S1 (S1) and stated as follows.

#### Drying by concentration (S1.1)

Open 1.5 mL tubes containing the SmMIT-LAMP reagents were subjected to vacuum in a Concentrator Plus (EPPENDORF, Germany) while centrifuged (V-AQ mode) at 1400 rpm at room temperature (RT). This action was carried out using the following drying procedures:

- **Two-step dry-up** (**S1**.**1a**). First, a mix containing 1.8 μL of primers, 0.56 μL of trehalose 2M (SIGMA, USA) and 0.14 μL of 50% glycerol was placed in the bottom of the tubes and concentrated for 30 min thus obtaining a dry pellet. Next, a mix containing 3.5 μL dNTPs, 1 μL *Bst* polymerase and 1.5 μL of trehalose (2M) was incorporated on top of pellet to be concentrated for additional 15 min. For the LAMP reaction, reconstitution of pellets was performed in 2.5 μL of buffer, 1.5 μL of MgSO_4_ and 19 μL of water (and in 2 μL of DNA template, if applicable) mixed up to a resulting final volume of 25 μL.
- **One-step dry-up** (**S1**.**1b**). A mix containing 1.8 μL of primers, 3.5 μL of dNTPs, 1 μL of *Bst* polymerase, 2.06 μL of trehalose and 0.14 μL of 50% glycerol was placed in the bottom of the tubes and concentrated for 30 min. Rehydration of the formed pellets was performed as indicated in the previous section (a).
- **All in one-step dry-up** (**S1**.**1c**). All necessary SmMIT-LAMP reagents were placed in the bottom of the tubes in the presence of 2 μL of trehalose (no glycerol) and then concentrated for a single step of 30 min. For later rehydration, only water up to a final volume of 25 μL was added (and 2 μL of DNA template, if applicable).

#### Drying by desiccation (S1.2)

This procedure was performed in all in one-step dry-up. Open 1.5 mL tubes containing all necessary SmMIT-LAMP reagents placed in the bottom in the presence of 2 μL of trehalose (no glycerol) were exposed to vacuum in a Concentrator Plus (EPPENDORF, Germany) without centrifugation (D-AQ mode) at RT for 30 min. For subsequently reaction, only water up to a final volume of 25 μL was added (and 2 μL of DNA template, if applicable).

To carry out the stabilized real-time SmMIT-LAMP tests, drying was performed in all in one-step desiccation procedure (that is, without centrifuge) in open 8-tube Genie Strips (OPTIGENE, UK) with or without the pre-addition of EvaGreen 20X (0.40 μL) in water to the LAMP master mixes (Supplementary Fig. S2). In some trials, DNA from *S*. *mansoni* was mixed with the other reagents from the beginning of the desiccation procedure to be used as ready-to-use positive control in tests. For later real-time reactions, the pellets were reconstituted in different volumes depending on the reagents included in the pre-desiccation process: (*i*) 25 μL water when including all LAMP components (counting with *S*. *mansoni* DNA); (*ii*) 23 μL water and 2 μL DNA template of *S*. *mansoni*; and (*iii*) 22.6 μL water, 2 μL DNA template of *S*. *mansoni* and 0.40 μL EvaGreen. After reconstituting the dried pellet by gently pipetting on the bottom of the tubes, the reaction was carried out in a portable Genie III device. In some assays, only 4/8-tube strips were prepared with dried reagents, thus keeping 4 tubes free to use with fresh liquid LAMP mixes to compare results running the same amplification real-time assay.

All SmMIT-LAMP and real-time SmMIT-LAMP dried reactions were carried out in a heat block and in a Genie III device, respectively, at 65 °C for 120 min followed up by 10 min at 80 °C to stop amplification. Amplification products were visualized with naked eye by adding 2 μL of SYBR Green I 1000× (INVITROGEN) in each post-amplified tube, by fluorescence monitoring and, by electrophoresis when required.

### Storage stability of dry-reagent LAMP mix

To estimate the stability and functionality over time, desiccated SmMIT-LAMP mixtures were stored at RT and 4 °C in cardboard storage boxes with some Silica Gel desiccant pouches inside to protect against moisture until use. After rehydration of the pellets, the LAMP reactions were performed on a heat block at 1 week, 3 weeks, 1 month, 3 months and 5 months.

## Results and Discussion

### Comparison of conventional and real-time SmMIT-LAMP assays

**Figure 1:**
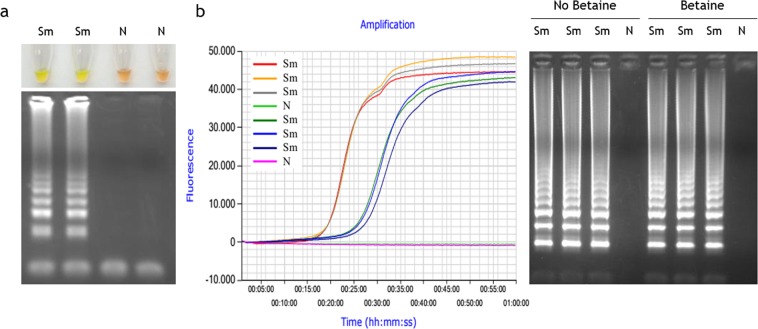
Comparison between conventional and real-time SmMIT-LAMP assays. (**a**) Conventional SmMIT-LAMP. Visual assessment based on addition of SYBR Green I after amplification (top) and electrophoresis on agarose gel (bottom). (**b**) Real-time SmMIT-LAMP. Time-course of EvaGreen 20X fluorescence signal over time (left) and electrophoresis results of free-betaine and 1 M betaine SmMIT-LAMP reactions (right). Sm, positive control (*S*. *mansoni* DNA; 1 ng); N, negative control (ultrapure water, no DNA). All electrophoresis gel images were obtained with an exposure between 700 ms and 1.2 s in a Gel documentation system, UVItec, UK. Real-time graph is directly captured from Genie Explorer Software (V2.0.5.5) result report.

SmMIT-LAMP result comparison between conventional and real-time conditions is shown in Fig. 1. Endpoint results at 65–70 min (counting 60 min for reaction plus 5–10 min of inactivation) were clearly observed with naked eye by adding the fluorescent dye SYBR Green I post-amplification. The positive LAMP reactions turned to green; otherwise, they remained orange. Correlation of positive colorimetric results with the typical ladder of multiple bands on agarose gels was clear (Fig. 1a). Real-time SmMIT-LAMP was carried out on a portable device using the same reaction conditions but testing including or not betaine in the master mix. Real-time reactions worked properly in both cases and a strong correlation between the signal of the fluorescent EvaGreen dye and electrophoresis was obtained (Fig. 1b). However, removing betaine from the mixture resulted on a 10 min reduction in the amplification time while showing identical intensity of electrophoresis bands. These findings were consistent with recent observations described by Ma *et al*.^37^ that associate an increased efficiency of the real-time LAMP with betaine-free conditions. Several mechanisms of action have been proposed for betaine, as increasing DNA accessibility^38^, preventing secondary structure formation in GC-rich region^39^ or pH dependent ion exchange^40^. Nevertheless, other studies consider that betaine has no effect at certain concentrations on the effectiveness of the LAMP assay^41,42^. In our study, 1 M betaine had no negative effect on real-time SmMIT-LAMP amplification, but betaine-free assay was faster, so all reactions from then on were performed without betaine.

### Stabilized conventional LAMP assay

**Figure 2:**
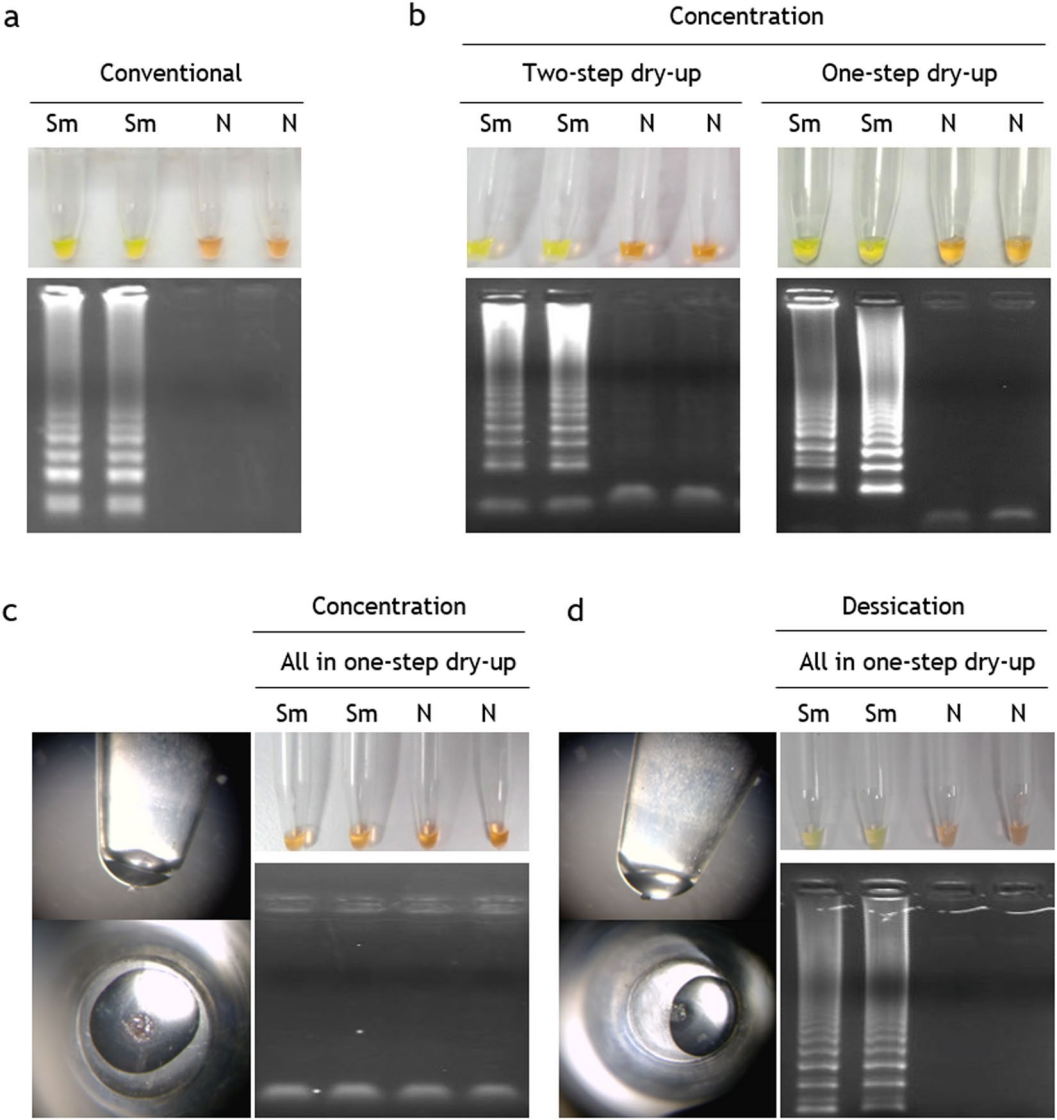
Operation of conventional SmMIT-LAMP and dry SmMIT-LAMP. (**a**) Results of conventional SmMIT-LAMP by color change and electrophoresis. (**b**) Results of SmMIT-LAMP after drying reagents by concentration following two-(left) and one-(right) step dry-up. (**c**) Results of SmMIT-LAMP after drying reagents by concentration following all in one-step dry-up. The pellet at the bottom of the tube obtained by 30 min concentration at RT is shown on the left. (**d**) Results of SmMIT-LAMP after drying reagents by desiccation following all in one-step dry-up. The pellet at the bottom of the tube obtained by 30 min concentration at RT is shown on the left. Sm, positive control (*S*. *mansoni* DNA; 1 ng); N, negative control (ultrapure water, no DNA). Electrophoresis gel images shown are cropped from different gels. All were obtained with an exposure between 700 ms and 1.2 s in a Gel documentation system, UVItec, UK.

Both concentration and desiccation procedures yielded stable and well-adhered pellets at the bottom of the tubes. Subsequent reconstitution of the pellets by gently pipetting was performed and conventional LAMP reactions were run for 2 h. The incubation time was increased up to 2 h because we obtained better amplification results in preliminary tests working with stabilized mixes. Figure 2 shows the comparison of the results of conventional SmMIT-LAMP (Fig. 2a) and dry SmMIT-LAMP prepared with all different tested stabilization protocols: concentration, in two and one (Fig. 2b) or all-in-one (Fig. 2c) dry-up steps, and desiccation (Fig. 2d). Drying by concentration resulted in a successful amplification when the reaction buffer and supplementary MgSO_4_ were not included in the stabilized master mix, regardless the use of a two-step or a one-step drying protocol. The visible color change results and those on electrophoresis showing bands coincided (Fig. 2b). By contrast, the concentration procedure in all-in-one step (including the reaction buffer from the beginning of the drying procedure) failed and no amplification was obtained (Fig. 2c). A probable explanation for this could be related to the instability of the master mix, since during the drying process the *Bst* polymerase is exposed to a high salt concentration when it is mixed with the reaction buffer, which causes the enzyme to become destabilized. This hypothesis has been recently reported for Phi29 polymerase used in rolling circle amplification (RCA) technique, and the separation of buffer and other salts into a different drying step has also been proposed for increasing significantly stability of master mixes storage over time^43,44^. In line with those findings, in our study, the two-step concentration protocol lead to functional master mixes but one-step concentration (including reaction buffer) did not. In addition, the centrifugation process probably favoured the formation of salt crystals precipitates that ended up inhibiting the subsequent amplification. Notwithstanding the evidence, a two-step concentration protocol would increase the time and complexity of the stabilization procedure in comparison to the one-step concentration protocol. Thus, in order to avoid possible precipitation by centrifuging and attempt the stabilization in an easier one-step protocol, we tried to stabilize all necessary SmMIT-LAMP reagents in one-step dry up desiccation procedure without centrifugation. Proceeding in this way, the subsequent reconstitution of reagents worked well and amplification results were observed both by colour change and in agarose gels electrophoresis (Fig. 2d). By avoiding centrifugation procedure (and therefore, special rotor adapters in concentrator), the desiccation protocol allowed us to stabilize the SmMIT-LAMP reagents not only in individual tubes, but also in 8-tube strips to perform the reaction in real-time using the portable Genie III device. Furthermore, by simple desiccation, multi-well plates could be prepared with stabilized master mixes allowing a greater number of samples to be analysed using either thermal cyclers in well-equipped laboratories or simple ovens or stoves when performing in low-resources settings.

### Stabilized real-time SmMIT-LAMP assay

**Figure 3:**
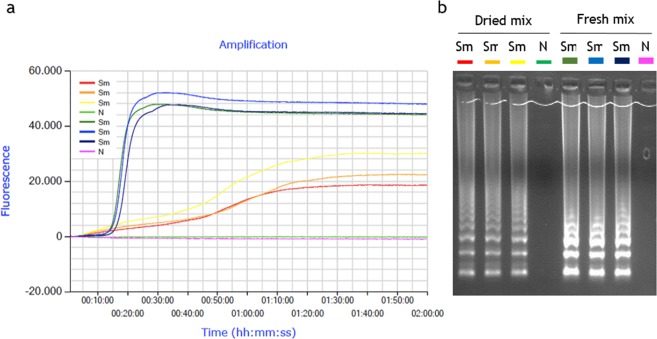
Operation of desiccation procedure to use in real-time SmMIT-LAMP. The assay was ran at 65 °C for a 2 h program in a portable Genie III device. (**a**) LAMP amplification with fluorescence detection with EvaGreen dye. (**b**) Agarose gel electrophoresis showing amplification results for both dried and fresh mixes. Sm, positive control (*S*. *mansoni* DNA; 1 ng); N, negative control (ultrapure water, no DNA). Real-time graph is directly captured from Genie Explorer Software (V2.0.5.5) result report.

The evaluation of desiccation procedure used in real-time SmMIT-LAMP is shown in Fig. 3. The 8-tube strips, containing four dried and four fresh liquid mixtures (operating controls), were incubated under isothermal conditions a 65 °C in a portable Genie III device. When reaction was running for 1 h (standard incubation time), only liquid mixtures yielded amplification results. A subsequent increase in reaction time (establishing a 2 h program) allowed us to obtained amplification also in dried mixtures, but later in time (aprox. 65–70 min *vs*. 20 min) and with a lower amplification level (30.000 *vs*. 50.000) than fresh liquid mixtures (Fig. 3a). Despite this, time-course of fluorescence during the reaction for dried mixtures was visibly detected and good quality amplification products appeared as typical ladder pattern on gel electrophoresis (Fig. 3b). A possible explanation for delay in DNA amplification after drying the reaction mixtures could be the role of trehalose in retarding conformational dynamics in dehydrated protein systems^45^, because in dried trehalose systems the guest molecules are homogeneously integrated into a hydrogen-bond network of water-trehalose which strongly restricts their mobility^46^. A probable restriction in mobility of *Bst* polymerase in a trehalose matrix at low water content could result into lower enzymatic activity and, therefore, in lower efficiency in DNA amplification.

### Stabilization of *S*. *mansoni* DNA for ready-to-use as positive control

**Figure 4:**
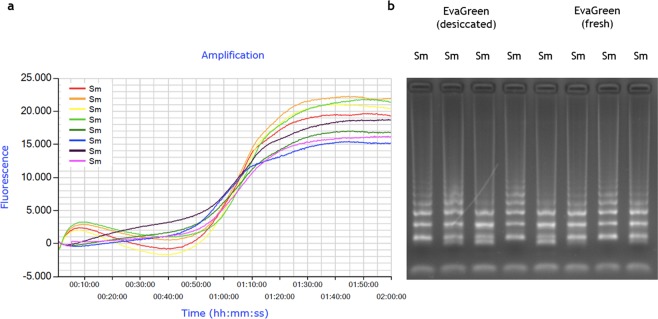
Results for real-time SmMIT-LAMP amplification using desiccated *Schistosoma mansoni* DNA as positive control ready-to-use. The assay was ran at 65 °C for a 2 h program in a portable Genie III device. (**a**) LAMP amplification with fluorescence detection with desiccated and fresh EvaGreen dye. (**b**) Agarose gel electrophoresis. Sm, positive control (*S*. *mansoni* DNA; 1 ng); Real-time graph was directly captured from Genie Explorer Software (V2.0.5.5) result report.

The desiccation procedure for real-time SmMIT-LAMP assays was also evaluated in 8-tube strips with mixes containing pre-incorporated *S*. *mansoni* genomic DNA (1 ng) as positive control ready-to-use for further diagnostic purposes (Fig. 4). Besides, EvaGreen^®^ dye was included or not in desiccation protocol. Next, dry-reagent LAMP mixes were reconstituted by adding just water (to those mixes containing *S*. *mansoni* DNA and dye) or water and fresh EvaGreen (to those mixes containing *S*. *mansoni* DNA but not dye). After 2 h of incubation, positive results were clearly observed by electrophoresis for both types of mixes, although with differences in fluorescence readings. The two types of dried mixes (with or without dried DNA-binding dye) yielded the same results in gel electrophoresis (Fig. 4b), but atypical small fluorescence readings appeared at the initial stages of the reactions when EvaGreen was previously dried (Fig. 4a). This could suggest a possible pre-amplification interaction between dye and DNA of *S*. *mansoni* during the desiccation process that ended up in a modification of the fluorescence signal during the reaction. The absence of information on this event in the literature does not allow us to compare our results, but we speculate with the possibility that this effect might be caused by an increase in the concentration of reagents after drying. The desiccation process would favor the interaction between dye and DNA, shifting the reaction equilibrium towards the bound conformation of EvaGreen and dsDNA, resulting in a small fluorescence signal^47^. This small fluorescence peak would disappear when the reagents become rehydrated and the reaction develops.

### Evaluation of the stabilized real-time SmMIT-LAMP assay on patients’ tissue samples

**Figure 5:**
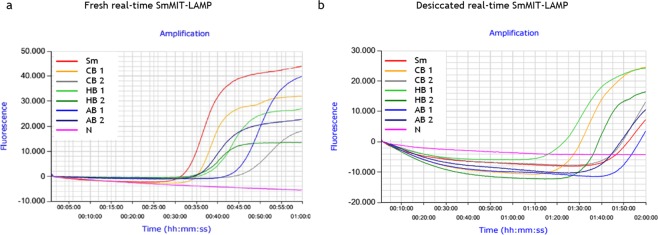
Amplification curves for *S*. *mansoni* in patients’ tissue samples using fresh and desiccated real-time SmMIT-LAMP mixtures. Outputs from the LAMP run using (**a**) fresh master mixes and (**b**) desiccated master mixes. Tissue samples were analyzed in duplicate (1 and 2); CB1, CB2: cutaneous biopsy; HB1, HB2: hepatic biopsy; AB1, AB2: appendix biopsy. Sm, positive control (*S*. *mansoni* DNA; 1 ng); N, negative control (ultrapure water, no DNA). Real-time graphs are directly captured from Genie Explorer Software (V2.0.5.5) result report. Sample names in the legend were manually added to the image.

DNAs from three patients’ tissue samples (skin, hepatic and appendix) with parasitological confirmed *S*. *mansoni* infection were used as “true samples” to test the potential applicability of the dry-reagent SmMIT-LAMP as ready-to-use test for schistosomiasis diagnosis (Fig. 5). To verify and compare results, a conventional real-time SmMIT-LAMP was also assessed (Fig. 5a). It is known that real-time SmMIT-LAMP works well with clinical samples, since we recently used it on patient’s skin biopsies to confirm the diagnosis of ectopic cutaneous schistosomiasis^48^. Both fresh and desiccated SmMIT-LAMP mixtures yielded amplification signals for *S*. *mansoni*-positive control and tissue samples. Nevertheless, a delay in the appearance of positive results and a decrease in the fluorescence signals were observed when using desiccated mixtures (Fig. 5b). As already discussed, this result would be in accordance with the potential restriction in mobility of *Bst* polymerase in a trehalose matrix at low water content, resulting in lower efficiency in DNA amplification.

### Stability performance over time of desiccated SmMIT-LAMP for conventional assays

**Figure 6:**
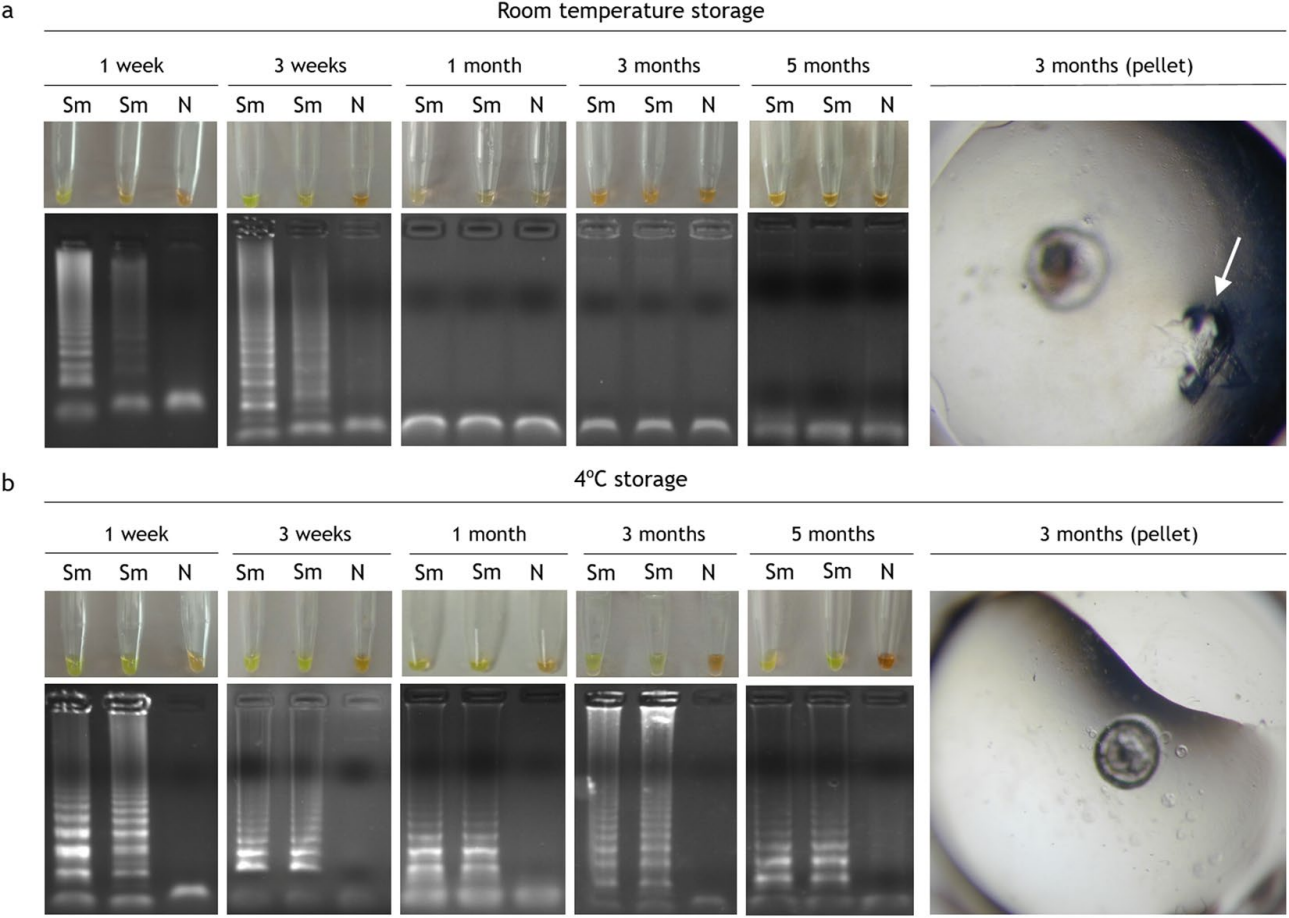
LAMP results of stabilized mixes over time at room temperature and 4 °C storage. (**a**) Room temperature storage. Visual inspection of reaction tubes positive (green) and negative (orange) and gel electrophoresis of DNA products. On the right, a pellet with a precipitate of crystals (white arrow) after 3 months storage at RT. (**b**) 4 °C storage. Visual inspection of reaction tubes positive (green) and negative (orange) and gel electrophoresis of DNA products. On the right, a pellet without any precipitate of crystals after 3 months storage at 4 °C. All electrophoresis gel images were obtained with an exposure between 700 ms and 1.2 s in a Gel documentation system, UVItec, UK. All gel images were captured within a day of the performance of the corresponding LAMP reaction.

The performance of the dry-reagent LAMP mix was assessed periodically for conventional assays by rehydration at one week, 3 weeks, one month, 3 months and 5 months (Fig. 6). Storage of dry-reagent LAMP mix at RT was found to be functional for 3 weeks (Fig. 6a). Remarkably, the dry-reagent LAMP mix proved to be stable up to 5 months at 4 °C (Fig. 6b).

A number of studies have attempted to address the problem of stabilizing molecular reagents for LAMP to be stored outside of the cold-chain, aiming to be applied for diagnostic purposes in field settings. Thus, different pre-mixed LAMP assays have been developed using lyophilization^22,23,49^, drying^21,26,50^ or combining different procedures, such as previous deglycerolization of *Bst* polymerase, followed by lyophilization of LAMP reagents^51^. Most studies highlight the proper operation of the dry-LAMP developed, but there are a number of differences and inaccuracies regarding the stability and functionality of the dry-reagent LAMP mix over time. Thus, in a study detecting *Leptospira* DNA, lyophilized reagents are stable for nearly 3 months when stored at 4 °C and 1 month when stored at 25 °C^49^. In another study, a dehydrated reagent mixture obtained by lyophilisation for detection of Zaire Ebola RNA Virus remained stable at RT for nearly 60 days with little loss of activity^22^. In the work of Howson *et al*.^23^, a lyophilized RT-LAMP for specific detection of foot-and-mouth virus was developed; in this case, although lyophilisation of reagents had no impact on the performance of the assay when compared to the equivalent “wet” reagents, no information about functionality over time is presented or discussed. On the other hand, several other studies on drying and stabilization of LAMP reagents using trehalose as cryoprotectant to prolong shelf-life at ambient temperature, have been published for detection of *Trypanosoma brucei rhodesiense*^21^, both *Plasmodium falciparum* and non-*P*. *falciparum*^26^ and *Trypanosoma evansi*^50^. In the study of Hayashida *et al*.^21^, the sensitivity of the dry RIME-LAMP -for detection of the repetitive insertion mobile element (RIME) target in *T*. *b*. *rhodesiense*) did not change after a 7-month storage at RT. However, with the same methodology, later used for the stabilization of the LAMP reagents for detection of *Plasmodium* spp.^26^ or *T*. *evansi*^50^, no information regarding functionality over time is disclosed. Finally, in the study of Engku Nur Syafirah *et al*.^51^, the dry-reagent LAMP mix developed for detection of toxigenic *Vibrio cholerae*, was found to be stable for 1 month at 4°C, 25°C and 37 °C; in addition, using the Q_10_ method, the shelf-life for this dry-LAMP was estimated at least 90.75 days at 25 °C.

In our work, the dry-reagent LAMP mix for *S*. *mansoni* detection resulted noticeably less stable over time at RT than the dry LAMP for *T*. *b*. *rhodesiense* detection developed by Hayashida *et al*.^21^ (3 weeks *vs*. 7 months, respectively). Probably, our attempt to dry the reagents in a single step was detrimental to the stability over time because of the accumulation of trehalose crystals in the dry pellets (Fig. 6a; white arrow). As discussed in previous reports^43,44^, under RT storage conditions, the presence of the reaction buffer in the stabilized reagent mix may favor crystallization of trehalose disrupting the oxidation barrier formed by the disaccharide and hindering the three-dimensional structure of the polymerase, thus resulting in a decrease or total loss of functionality. Despite this limitation, maintaining the functionality for at least 3 weeks at RT would allow us to prepare and distribute a set of dried SmMIT-LAMP master mixes to be used within a few weeks in field surveys of schistosomiasis in resource-limited areas. Further studies using other stabilizers are needed to achieve a better thermal stability of the LAMP reagents at ambient temperature. In this way, a new simple method that uses pullulan or mixture of pullulan and trehalose to achieve the long-term stabilization of LAMP master mixes has been recently reported^44^.

By contrast, no crystallization was observed when storing the dry-reagent LAMP mix at 4 °C (Fig. 6b), thus likely maintaining the functionality of the mixture until at least 5 months of storage. To the best of our knowledge, this is the longest period of storage of stabilized LAMP reagents at 4 °C while maintaining functionality. Although our stabilized SmMIT-LAMP assay require a cold storage, its ready-to-use features have improved the previous conventional LAMP, reducing the possibilities of cross contamination during multiple pipetting steps in master mix preparation and repeated freezing and thawing of reagents prior use.

Point-of-care testing (POCT) is defined as laboratory diagnostic testing performed at or near the site where clinical care is delivered, allowing a rapid outcome and, potentially, the application of an early treatment^52^. The ideal POCT should be user-friendly and as simple as possible so that, even those without technical or clinical knowledge, would be able to use it and understand its response^53^. The ASSURED (Affordable, Sensitive, Specific, User-friendly, Robust and Rapid, Equipment-free and Deliverable) criteria established by the WHO, is a general benchmark to work towards developing POCT diagnosis^54^ and it needs to be revisited for LAMP application in endemic areas. LAMP technology is a field-friendly alternative to many other complex molecular methods^55^ and it has been proposed as an ideal candidate for revolutionizing point-of-care molecular diagnostics. In addition, novel LAMP-based platforms have been developed for pathogen detection and significant improvements have been made also in monitoring LAMP results, both end-point and real-time, thus bringing closer the LAMP technology to a realistic point-of-care format for resource-poor endemic areas^56^. The simplification, both in the number of steps and the infrastructure needed, would be a great advance in manufacturing and lowering the cost of preparation.

In summary, we present here the development and application of a novel and simple desiccation procedure for drying LAMP reagents using trehalose that can be also adapted for conventional and real-time amplification assays. The one-step dry-up protocol is simpler and faster than those previously reported and allows to maintain the functionality for at least 3 weeks at RT and up to 5 months at 4 °C. Our work demonstrates an important improvement for SmMIT-LAMP molecular assay, transformed into a cold maintenance dry format suitable for manufacturing as kit for ready-to-use for *S*. *mansoni* DNA detection. This procedure could be applied to create ready-to-use molecular dry LAMP kits for a laboratory setting and for POC diagnostic applications for several other NTD. Optimization and improvement of long-term stability at ambient temperature for a real application as a POCT in field conditions is still needed and currently ongoing.

## Supplementary information

Supplementary information

## Data Availability

No additional data (other than stated in the manuscript) was produced or used for the preparation of the manuscript. All data generated or analysed during this study are included in this published article (and its Supplementary Information files).
