# Supplementary material for: Progress in loop-mediated isothermal amplification assay for detection of Schistosoma mansoni DNA: towards a ready-to-use test
Source: Sci Rep. 2019 Oct 14;9:14744. doi: 10.1038/s41598-019-51342-2 (PMC6791938; doi:10.1038/s41598-019-51342-2)
Supplement: Supplementary file 1 — Supplementary information [file 41598_2019_51342_MOESM1_ESM.docx]

**Progress in loop-mediated isothermal amplification assay for detection of *Schistosoma mansoni* DNA: towards a ready-to-use test.**

García-Bernalt Diego, J.^1^, Fernández-Soto P*.^1^, Crego-Vicente B. ^1^, Alonso-Castrillejo S. ^1^, Febrer-Sendra B. ^1^, Gómez-Sánchez A. ^1^, Vicente B. ^1^, López-Abán J. ^1^, Muro A*^1^.

^1^Infectious and Tropical Diseases Research Group (e-INTRO), Biomedical Research Institute of Salamanca-Research Centre for Tropical Diseases at the University of Salamanca (IBSAL-CIETUS), Faculty of Pharmacy, University of Salamanca, Salamanca, Spain.

***Corresponding authors:**

Pedro Fernández-Soto, [pfsoto@usal.es](mailto:pfsoto@usal.es)

Antonio Muro Álvarez, [ama@usal.es](mailto:ama@usal.es)

**Supplementary Information**

**Table of contents**

**Supplementary Figure 1 (S1).** Stabilization procedures of SmMIT-LAMP reagents for conventional assays……………………………………………………………………...2

**Supplementary Figure 2 (S2).** Stabilization procedures of SmMIT-LAMP reagents for real-time assays………………………………………………………………………….3

**Supplementary Figure 1**


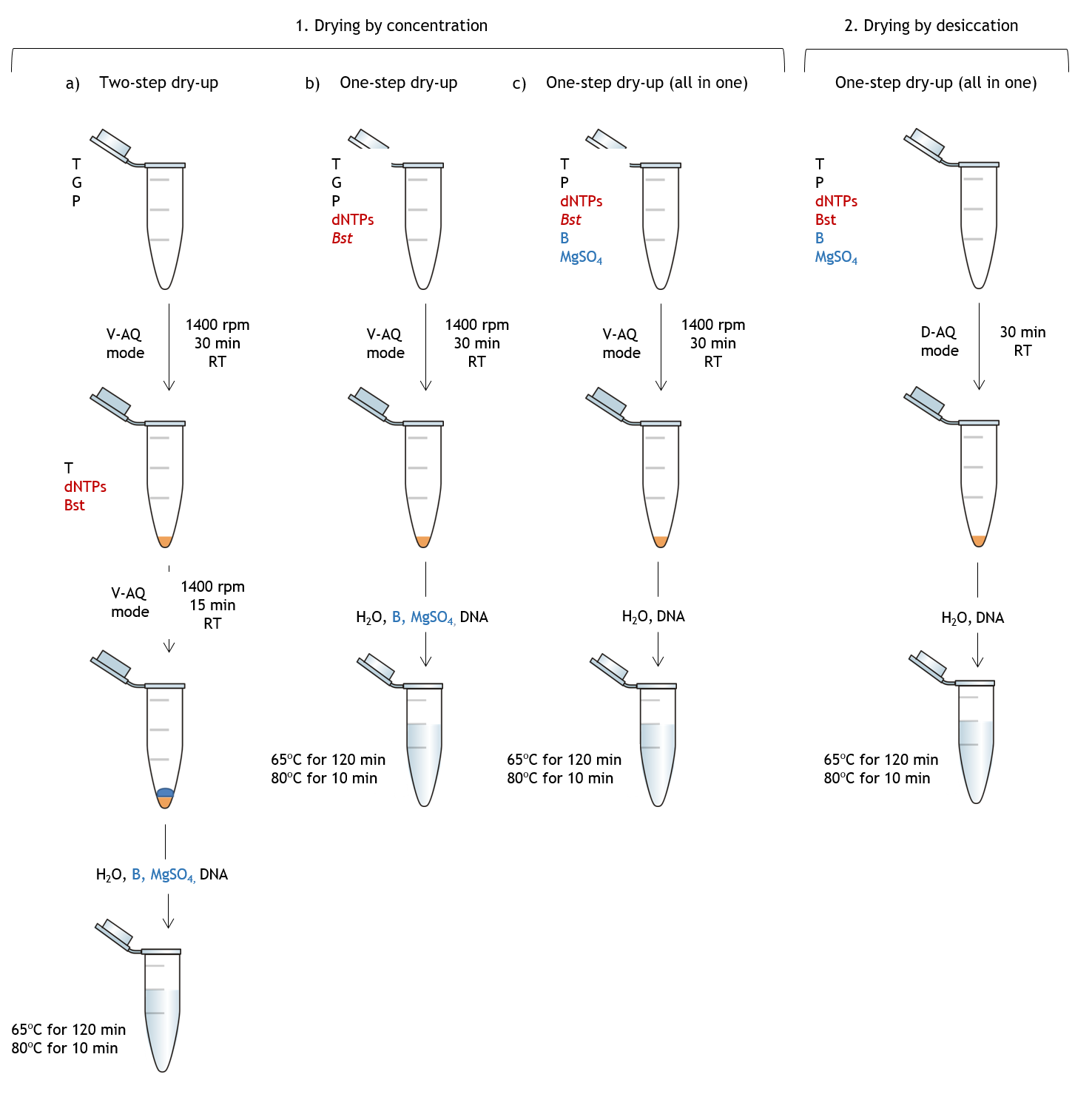


**Supplementary Figure 1 (S1). Stabilization procedures of SmMIT-LAMP reagents for conventional assays.** 1. Drying by concentration. (1a) Concentration protocol following two dry-up steps. (1b) Concentration protocol following one dry-up step. (1c) Concentration protocol following one dry-up step (all in one). 2. Drying by desiccation using one dry-up step (all in one). T, trehalose; G, glycerol; P, primers; dNTPs, deoxynucleotides; Bst, *Bst* polymerase 2.0 WarmStart; B, isothermal buffer; MgSO_4_, magnesioum sulphate; DNA, deoxyribonucleic acid; V-AQ mode, with centrifugation; D-AQ, without centrifugation; RT, room temperature; rpm, revolutions per minute.

**Supplementary Figure 2**


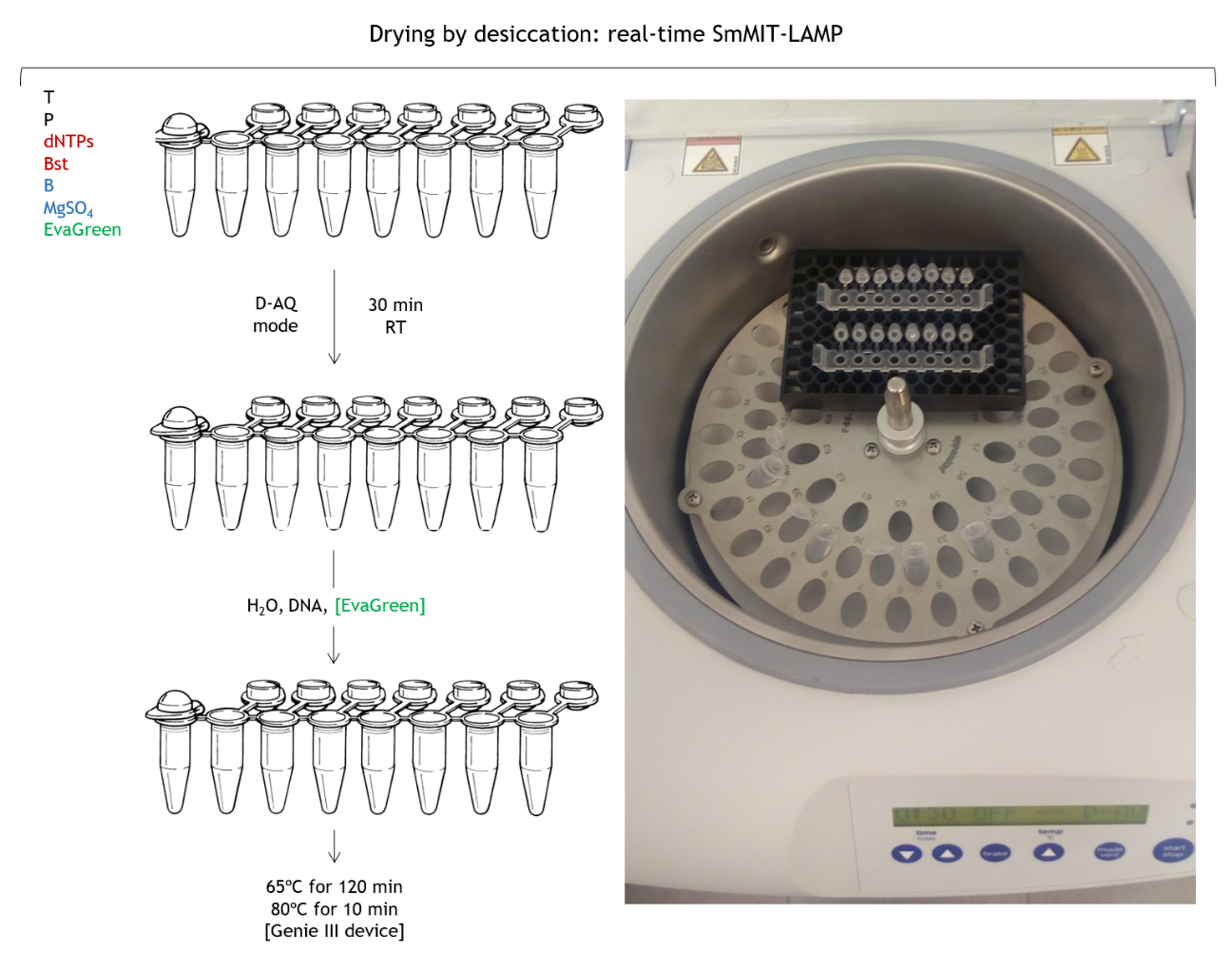


**Supplementary Figure 2 (S2). Stabilization procedures of SmMIT-LAMP reagents for real-time assays.** Drying was performed in all in one-step desiccation procedure in open 8-tube strips with or without the pre-addition of EvaGreen dye. T, trehalose; P, primers; dNTPs, deoxynucleotides; Bst, *Bst* polymerase 2.0 WarmStart; B, isothermal buffer; MgSO_4_, magnesium sulphate; EvaGreen (included in pre-desiccation), D-AQ, without centrifugation; RT, room temperature; rpm, revolutions per minute; DNA, deoxyribonucleic acid; [EvaGreen], added in rehydration step.
